# Heart surgery over two decades: what we have learned about results and changing risks

**DOI:** 10.1186/s12872-024-03860-9

**Published:** 2024-04-05

**Authors:** Masih Tajdini, Amir Hossein Behnoush, Mina Pashang, Mana Jameie, Amirmohammad Khalaji, Saeed Sadeghian, Ali Vasheghani-Farahani, Hamidreza Poorhosseini, Farzad Masoudkabir, Kaveh Hosseini, Saeed Davoodi, Mohammad Sahebjam, Khosro Barkhordari, Haleh Ashraf, Akbar Shafiei, Abbasali Karimi, T. H. C. Surgery Databank Collaborators, Seyed Hossein Ahmadi Tafti, Soheil Mansourian, Mahmood Shirzad, Jamshid Bagheri, Arash Jalali, Kiomars Abbasi, Arezou Zoroufian, Ali Hosseinsabet, Tahereh Davarpasand, Reza Mohseni-Badalabadi, Reza Hali, Mohammadjavad Mehrabanian, Mehdi Dehghani Firoozabadi, Behrang Nooralishahi, Seyedeh Hamideh Mortazavi, Masoumeh Lotfi-Tokaldany, Elham Rostami, Mahdieh Karimi

**Affiliations:** 1 Tehran Heart Center, Cardiovascular Diseases Research Institute, Tehran University of Medical Sciences, Tehran, Iran; 2 Cardiac Primary Prevention Research Center, Cardiovascular Diseases Research Institute, Tehran University of Medical Sciences, Tehran, Iran

**Keywords:** Cardiac Surgery, Developing Countries, Iran, Tehran Heart Center, Trend

## Abstract

**Objectives:**

Using the cardiac surgery database is of high importance in referral centers and can lead to a better quality of care for patients. Tehran Heart Center (THC) is a cardiovascular referral center that was inaugurated in 2001. In this report, we aimed to present the third report of trends in patients' cardiovascular risk factors and surgical procedures from 2002 to 2021 that have been gathered for all THC patients.

**Methods:**

This** s**erial cross-sectional study was conducted at Tehran Heart Center from 2002 to 2021. All patients undergoing cardiac surgeries were eligible to enter the study (*N* = 63,974). Those with miscellaneous types of surgeries were excluded (*N* = 9556). The distribution of cardiac surgeries (including isolated coronary artery bypass graft (CABG), isolated valve, and CABG + valve surgeries) and their respective in-hospital mortality were recorded. Furthermore, 20-year trends in the prevalence of various cardiovascular risk factors (CVRFs) among the following groups were evaluated: a) isolated CABG, b) aortic valve replacement/repair for aortic stenosis (AS/AVR/r), and c) isolated other valve surgeries (IVS).

**Results:**

A total of 54,418 patients (male: 70.7%, age: 62.7 ± 10.8 years) comprised the final study population, with 84.5% prevalence of isolated CABG. Overall, the AS/AVR/r group was in between the CABG and IVS groups concerning CVRFs distribution. Excluding some exceptions for the AS/AVR/r group (in which the small sample size (*N* = 909) precluded observing a clear trend), all studied CVRFs demonstrated an overall rising trend from 2002 to 2021 in all three groups. Regarding in-hospital mortality, the highest rate was recorded as 4.0% in 2020, while the lowest rate was 2.0% in 2001.

**Conclusions:**

Isolated CABG remained the most frequent procedure in THC. Notable, increasing trends in CVRFs were observed during this 20-year period and across various types of cardiac surgeries, which highlights the clinical and policy-making implications of our findings.

**Graphical Abstract:**

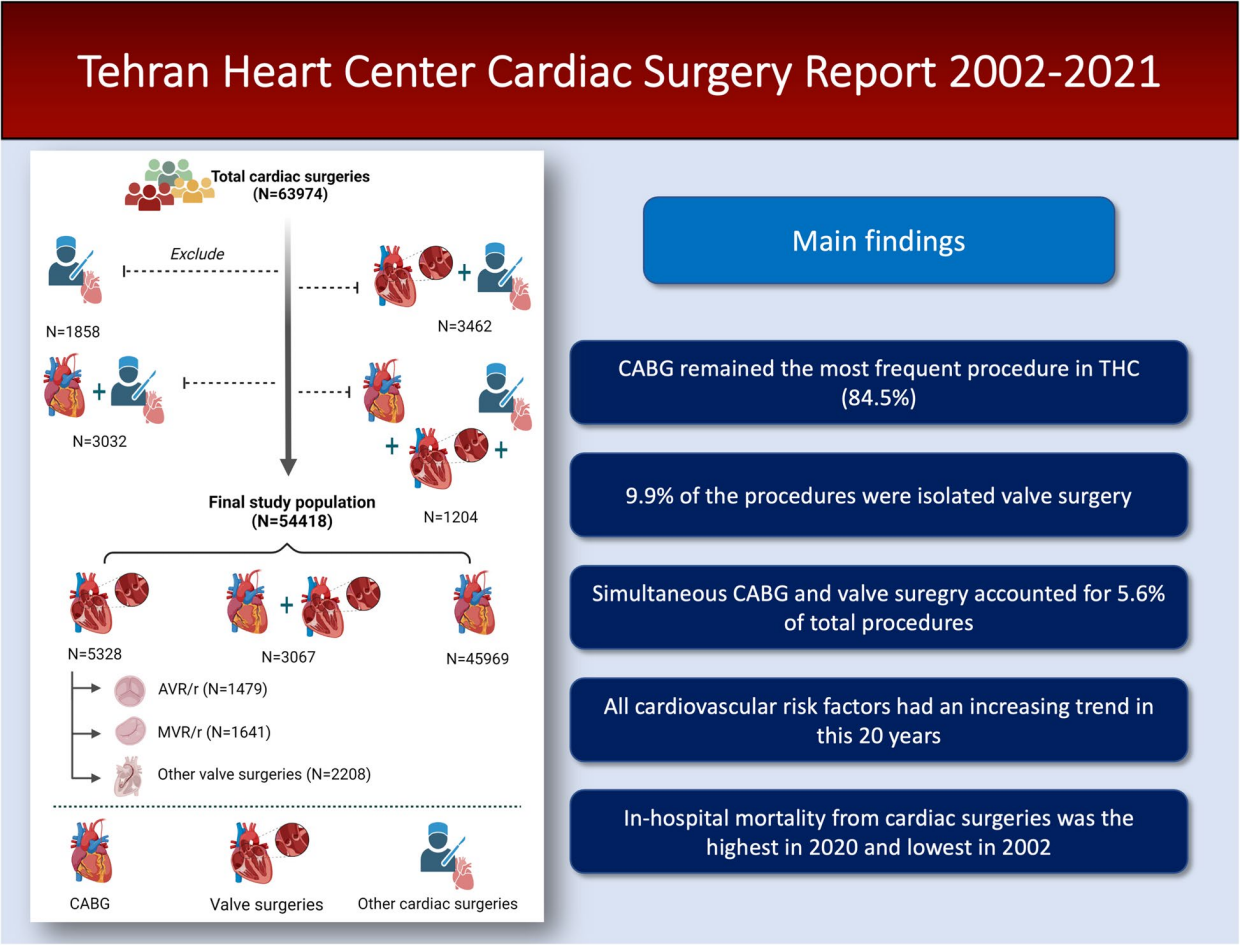

**Supplementary Information:**

The online version contains supplementary material available at 10.1186/s12872-024-03860-9.

## Introduction

In the era of rapid exchange of medical knowledge, healthcare databases are considered the mainstay of progress toward a better quality of care. Healthcare knowledge management, first introduced in 1995, can lead to cost reduction and innovation as well. Surgical procedure monitoring can provide several benefits, including usage appropriateness assessment, efficacy and safety evaluation, community trends estimation, and healthcare cost calculation [1, 2]. This issue has been perennially regarded as highly important in the field of cardiothoracic surgery, as evinced by the establishment of the first cardiac surgery national database in 1986 by the Society of Thoracic Surgeons (STS). This Adult Cardiac Surgery Database (ACSD) is now comprised of more than 7.4 million records of patients all across the United States [3]. Similarly, the European Association for Cardio-Thoracic Surgery (EACTS) Adult Cardiac Database (ACD) reports data from more than 80 European hospitals and 120,000 surgical interventions [1], highlighting the importance of developing regional databases in other countries as well.

Tehran Heart Center (THC) is a dedicated cardiovascular referral center affiliated with the Tehran University of Medical Sciences and was inaugurated in 2001 [4]. Database collection has been started in THC since then with the goal of enhancement of care quality based on data from patients referring to THC for possible use at the national level. The THC ACSD database documents over 150 pre-operative, intra-operative, and post-operative variables and has enrolled more than 60,000 patients since 2002. With this plethora of patients' records, the THC research department is well-known for both basic science and clinical research projects. The last two reports on THC ACSD were published in 2008 [5] and 2012 [6].

Herein, we aimed to present the third report in which trends in different surgical procedures (types, characteristics, and in-hospital mortalities) and patients' cardiovascular risk factors from 2002 to 2021 have been gathered for THC patients undergoing various types of cardiac surgeries. This not only illustrates the trend and mortality of surgeries in a larger cardiac referral center in Iran that can have policy implications but could provide clinicians with updated risk factors and healthcare systems with localized trends in order to design better interventions.

## Materials and methods

### Study design and population

This cross-sectional registry-based study was conducted at Tehran Heart Center (THC) and aimed to examine the trends of cardiac surgeries and patients' cardiovascular risk factors from 2002 to 2021. THC hospital- affiliated with Tehran University of Medical Science, Tehran, Iran- is one of Iran's major tertiary referral cardiovascular canters, with a mean surgery volume of 3198 cases per year. The THC cardiac surgery databank encompasses more than 150 pre-operative, intra-operative, and post-operative variables [4].

The study complied with Helsinki principles and was approved by the Ethics Committee of Tehran Heart Center (IR.TUMS.MEDICINE.REC.1402.437). The need for informed consent was waived by the ethics committee due to the study's retrospective nature and anonymization of the data. The study was performed in line with the Strengthening the Reporting of Observational Studies in Epidemiology (STROBE) checklist for cross-sectional studies (Supplementary Materials).

### Study population

**Fig. 1:**
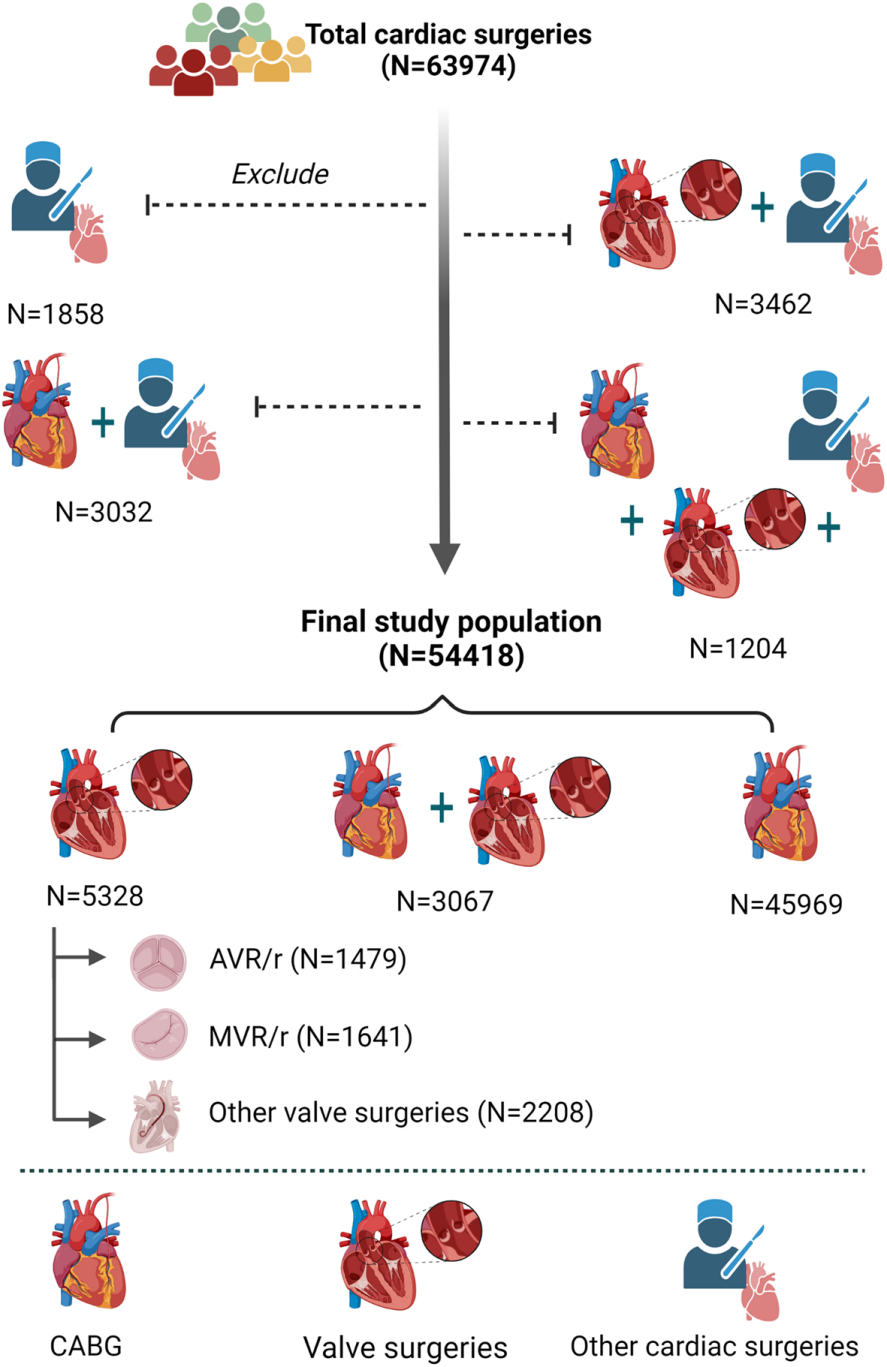
Study flowchart

Figure 1 depicts the flow chart of the study population. Patients undergoing cardiac surgeries from 2002 to 2021 were eligible to enter the study (*N* = 63,974). Patients who underwent any isolated or simultaneous cardiac surgeries rather than coronary artery bypass grafting (CABG) and valve surgeries were excluded (*N* = 9956). Accordingly, 54,418 patients comprised the final study population, including patients undergoing isolated CABG, isolated valve surgeries, and simultaneous CABG and valve surgeries ("CABG + valve" group).

For identifying the 20-year trends of cardiovascular risk factors across cardiac surgeries, patients were classified into three groups: a) CABG group; b) AS.AVR/r group: aortic valve replacement (AVR) or repair (AVr) surgeries for aortic stenosis (AS) due to higher burden and prevalence, compared with other valve surgeries [7]; c) IVS (isolated other valve surgeries): all other valve surgeries excluding AS.AVR/r. The rationale for such classification was to test the hypothesis whether the prevalence of risk factors among patients in the AS.AVR/r lies between the CABG and the IVS group.

### Variable definition

The variable definition was according to The Society of Thoracic Surgeons (STS) manual [8] and in line with our previous studies on this population [9, 10] (Supplementary Materials for more details).

### Statistical analyses

Categorical variables are presented as frequency (percentages) and were compared using the Chi-Squared test. Continuous variables are presented as mean ± standard deviation and were compared using the Independent Student's T-test. Using the "Graphing and Visualization" tool of Microsoft Excel software 2019, values of each variable of interest were demonstrated across the 20-year study period, and trend lines were constructed. The IBM SPSS Statistics for Windows, version 23.0 (Armonk, NY: IBM Corp) was used, and *P* values < 0.05 were considered significant.

## Results

### Twenty-year trends of cardiac surgeries

**Fig. 2:**
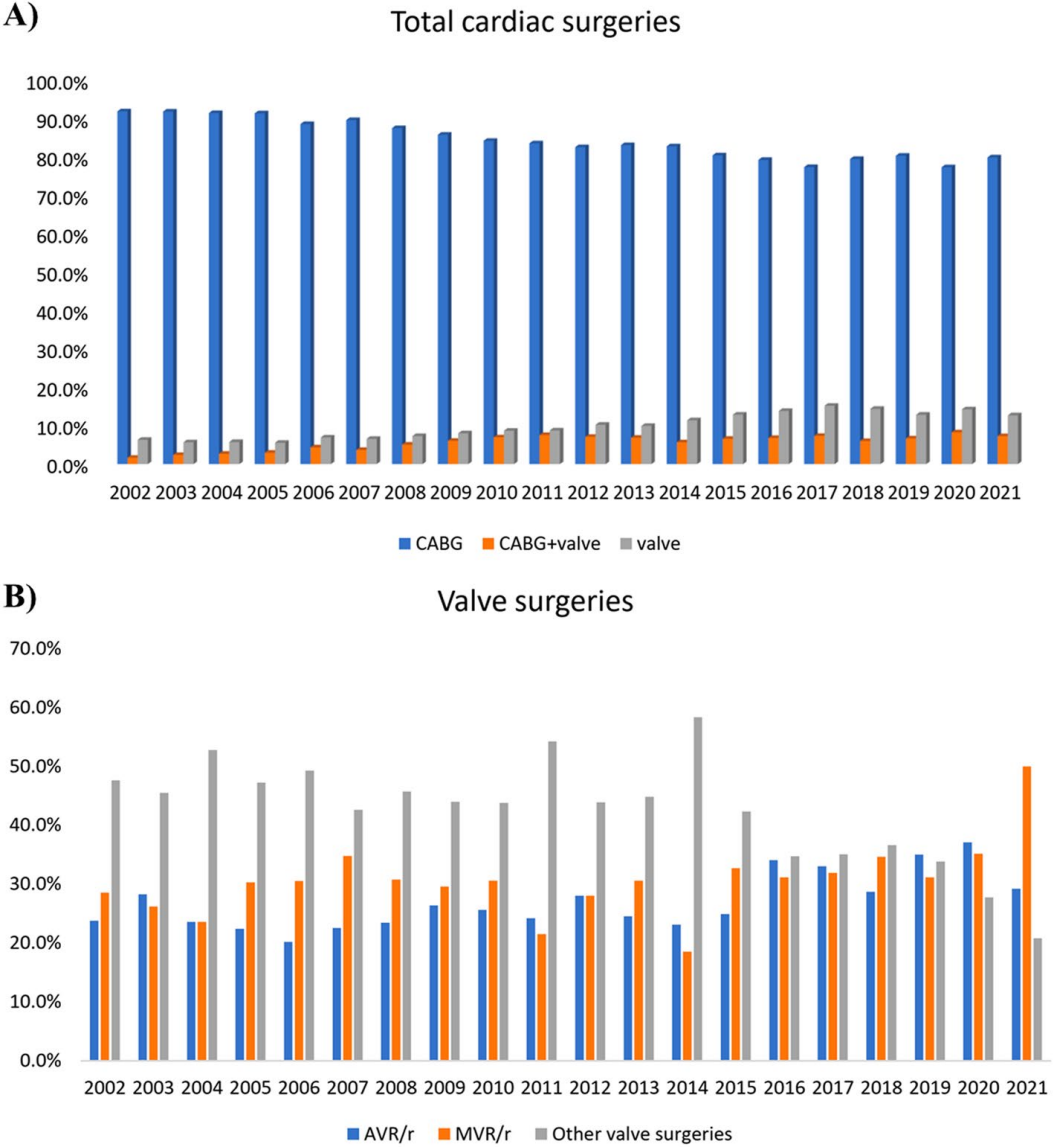
Twenty-year trends of cardiac surgeries **A**) Total and **B**) Valve surgeries

A total of 54,418 cardiac surgeries were conducted during 20 years, with isolated CABG being the most frequently performed surgery (*n* = 48,969, 84.5%), followed by isolated valve surgery (*n* = 5382, 9.9%) and simultaneous CABG + valve surgery (*n* = 3067,5.6%). The yearly frequency of total surgeries ranged from 1322 in 2002 to 3397 in 2008. The greatest number of isolated CABG, isolated valve, and CABG + valve surgeries were performed in 2004 (89.9%), 2017 (12.9%), and 2011 (6.3%), respectively (Fig. 2A, Supplementary Table 1). Among valve surgeries, a gradual decrease in other valve surgeries was observed with a reciprocal rise in both AVR/r and MVR/r surgeries (Fig. 2B). Supplementary Table 2 represents the number and proportion of valve surgeries conducted each year. The highest number of AVR/r was conducted in 2017, while this was in 2017 and 2018 for MVR/r. The two types of mitral valve surgeries fluctuated widely over the study period, with MVr surgeries varying from 0 to 23% and MVR from 78 to 100%, while AVR ranged from 92 to 100% and AVr from 0 to 8%. The percentage of each AVR/AVr and MVR/MVr are shown in Supplementary Tables 3 and 4, respectively.

#### Patient characteristics

Among the total of 54,418 patients (male: 70.7%, age: 62.7 ± 10.8), those who underwent CABG + valve surgeries were significantly older (65.82 ± 9.84 years old) with the highest prevalence of LVEF ≤ 40 (*n* = 10,321, 43.7%) compared to isolated CABG and isolated valve surgeries (Ps < 0.001). The CABG group was significantly more likely to have other cardiovascular risk factors, including diabetes (*n* = 17,386, 37.9%), hypertension (*n* = 24,839, 54.1%), hyperlipidemia (*n* = 26,314, 57.4%), current cigarette smoking (*n* = 8594, 18.7%), opium ever use (*n* = 5853, 14.9%), positive family history of coronary artery diseases (*n* = 16,351, 35.7), and overweight (*n* = 10,138, 23.3%) (all Ps < 0.001) (Table 1). Missing values of each variable of interest are demonstrated in Table 1.

**Table 1:** Baseline characteristics of patients (*N* = 54,418)

**Characteristic**	**Missing rate**	**CABG** *N* = 45,969	**CABG + valve** *N* = 3067	**Valve** *N* = 5382	***P***
Men	0%	34,066 (74.1%)	2024 (66.0%)	2394 (44.5%)	< 0.001
Age	0%	63.37 ± 10.33	65.82 ± 9.84	55.45 ± 14.30	< 0.001
Diabetes	0.3%	17,386 (37.9%)	1029 (33.8%)	668 (12.5%)	< 0.001
Hypertension	0.3%	24,839 (54.1%)	1584 (52.1%)	1609 (30.2%)	< 0.001
Dyslipidemia	0.4%	26,314 (57.4%)	1465 (48.1%)	1465 (27.5%)	< 0.001
Current cigarette smoking	0.4%	8594 (18.7%)	472 (15.5%)	431 (8.1%)	< 0.001
Positive IHD family History	0.5%	16,351 (35.7%)	841 (27.7%)	1,156 (21.7%)	< 0.001
Opium ever user	14%	5853 (14.9%)	413 (14.7%)	356 (7.4%)	< 0.001
LVEF ≤ 40	1.1%	13,485 (29.6%)	1321 (43.7%)	898 (17.0%)	< 0.001
BMI ≥ 30	6.1%	10,138 (23.3%)	591 (21.1%)	981 (20.2%)	< 0.001

Data are presented as frequency (percentages) or mean ± standard deviation

*CABG* coronary bypass graft, *IHD* ischemic heart disease, *LVEF* left-ventricular ejection fraction, *BMI* body mass index

### Twenty-year trends of cardiovascular risk factors

#### Gender

In contrast to the IVS group with a female predominance (60.4% female), isolated CABG and AS.AVR/r had predominantly male populations (74.1% and 65.9% male, respectively). The 20-year distribution of male and female patients remained relatively constant across all the study groups.

#### Age

**Fig. 3:**
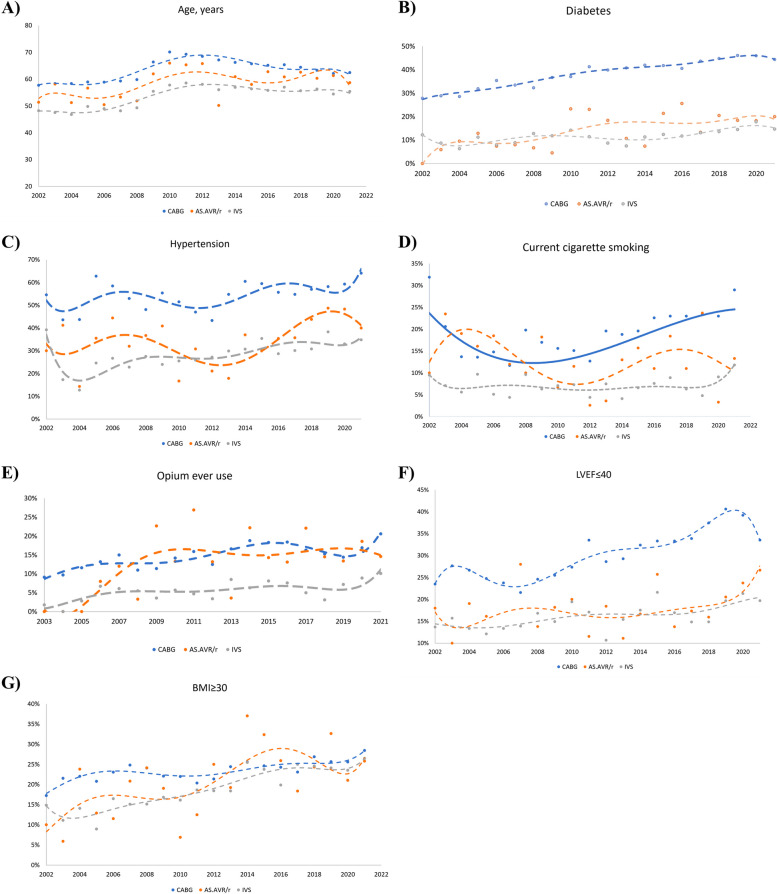
Twenty-year trends of cardiovascular risk factors according to cardiac surgery types **A**) Diabetes, **B**) Hypertension, **C**) Current cigarette smoking, **D**) Opium ever use, **E**) Age, **F**) LVEF ≤ 40, and **G**) BMI ≥ 30

CABG patients comprised the oldest study group (63.37 ± 10.33), followed by AS.AVR/r (59.7 ± 15.55) and IVS (54.55 ± 13.88). Generally, the population moved towards aging from 2002 to 2021, peaking in 2010–2012 in all the study groups. Particularly, the CABG and IVS groups followed a comparable pattern in that they underwent a period of an incrementing trend from 2006 to 2011, followed by a subsequent decrement until 2016. This decrease persisted until 2021 for the CABG group (Fig. 3A, Supplementary Table 5).

#### Diabetes

The total and yearly prevalence of diabetes was the highest in the CABG group (2002:27.8%, 2021: 44.4%. total: 37.9%). This total prevalence was more than twice as much as the AS.AVR/r group (16.1%) and more than three times as much as the IVS group (11.8%). Despite minor periodic fluctuations, particularly in the IVS group, diabetes prevalence experienced a steady upward trend across all the categories. The most prominent increase was documented in the AS.AVR/r group (2002–2021 difference: 20%, maximum-minimum difference: 25.6%); however, it could be due to the small number of patients in this group (Fig. 3B, Supplementary Table 5).

#### Hypertension

In line with diabetes, the yearly and total prevalence of hypertension was the highest among CABG patients (54.1%), followed by AS.AVR/r (36%) and IVS (28.9%) groups. Excluding some volatility from 2002–2005 and in 2021, the prevalence of hypertension went through an overall rising trend among all the study groups. This increase particularly intensified from 2013 onwards, resulting in more than 55%, 35%, and 30% of CABG, AS.AVR/r, and IVS patients experienced hypertension over most of the 2013–2021 period. This surge peaked in 2021 for the CABG group (64.1%) and in 2019 for the AS.AVR/r (48.7%) and IVS (38.3%) groups (Fig. 3C, Supplementary Table 5).

#### Current cigarette smoking

The majority of smokers belonged to the CABG group (18.7%), followed by AS.AVR/r (13.3%) and IVS (9.5%) groups. The same pattern was applied to the most prominent difference between maximum and minimum prevalence levels (CABG: 20.2%; AS-AVR: 16.1%; IVS: 7.7%). Apart from 2002–2003, smoking prevalence escalated during the study period; however, the rise was steadier and more regular from 2014 onwards. The prevalence mainly varied from 4 to 9% in the IVS group, with a more apparent increase from 2014 afterward. Due to prominent rises and falls in the AS.AVR/r category, no recognizable trend was observed in this group (Fig. 3D, Supplementary Table 5).

#### Opium ever use

Patients in CABG and AS.AVR/r groups had similar and higher rates of opium ever use, compared with the IVS group (CABG: 14.9%, AS.AVR/r: 14.6%, IVS: 5.3%). While no clear pattern was recordable for the AVR-AS group due to considerable volatility, CABG and IVS groups demonstrated a parallel pattern. The prevalence climbed overall in both groups (from 10.8% to 20.6% in the CABG and from 0% to 10.1% in the IVS group), with three discrete periods of decreases in 2008, 2012, and 2016–2017. Subsequently, the prevalence rose again during the last years of the study period, resulting in the highest prevalence in 2020–2021 (Fig. 3E, Supplementary Table 5).

#### LVEF ≤ 40

LVEF ≤ 40 was the most prevalent in the CABG group (29.6%) and was higher than both groups by approximately 10% (AS.AVR/r group prevalence: 19.4%; IVS group prevalence: 16.6%). The AS.AVR/r group had various rises and falls, precluding us from distinguishing a particular trend pattern. From 2002 to 2021, the overall prevalence of LVEF ≤ 40 escalated by 10% in the CABG and 6% in the IVS group, leading to a respective 2021 prevalence of 33.5% and 19.7%. The increasing trend was notably consistent between 2007–2019 for the CABG and 2005–2014 for the IVS group (Fig. 3F, Supplementary Table 5).

#### BMI ≥ 30 (obesity)

The obesity prevalence was the highest in the CABG group (29.6%), followed by AS.AVR/r (23.4%) and IVS groups (19.8%). Although all three groups underwent an overall climbing trend with a near 15% increase in the AS-AVR, 12% in the IVS, and 10% in the CABG group, the pattern differed moderately between the three groups. AS-AVR group saw various periods of instability with a generally higher prevalence in the second half of the study. Likewise, the mentioned higher prevalence in the second half applied to CABG and IVS patients; however, year-by-year increases in the CABG group were less prominent. Owing to a steeper increasing slope in the CABG compared to the IVS group, the two trend lines converged so much that obesity prevalence reached almost common levels in the last years (Fig. 3G, Supplementary Table 5).

### Twenty-year trends of In-hospital mortality

A total of 1046 (1.9%) in-hospital mortality occurred, the highest rate of which pertained to CABG + valve surgeries (7.2%), followed by isolated valve (3.5%) and isolated CABG surgeries (1.40%). Likewise, apart from a few exceptions (2002–2006 period and 2021), the annual mortality rate followed the same pattern as total mortality rates among the three groups.

**Fig. 4:**
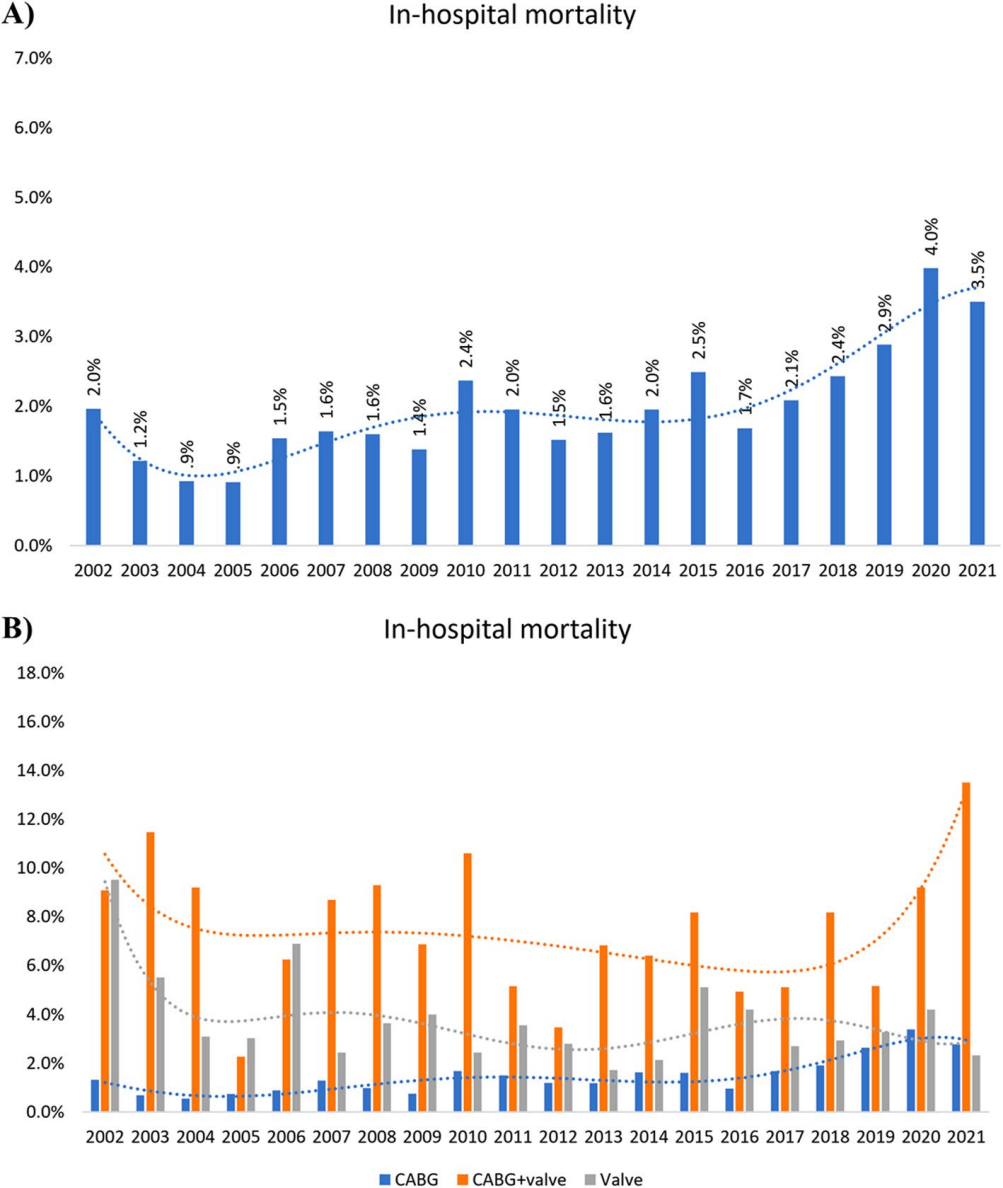
Twenty-year trends of in-hospital mortality among cardiac surgeries **A**) Total and **B**) Classified

Excluding two periods of decrement in 2003–2005 and 2011–2014, total in-hospital mortality varied slightly from 2.0% in 2001 to 4.0% in 2020 and 3.5% and 2021 (Fig. 4 and Supplementary Table 6). The in-hospital mortality rate in the CABG + valve group demonstrated a considerably erratic behavior (maximum-minimum difference: 11.2%), plausibly reflecting the small sample size in this group. Nevertheless, the rates were generally higher at both ends of the study period (peaks: 11.5% in 2003 and 13.5% in 2021) compared to that in the years between (plunges: 2.3% in 2005 and 3.5% in 2012). The isolated valve surgery group experienced volatile yet more stable changes than the CABG + valve group mortality, ranging from 1.7% to 9.8%. Mortality rates were sizably higher in the first two years and plummeted subsequently to less than 4.2% in the majority of the following years. Excluding 2002, the mortality rate in the CABG group remained almost stable and less than 1% until 2009, followed by minute increments from 2014 onwards (Supplementary Table 6).

## Discussion

Here we reported the trends of 54,418 cardiac surgeries, including isolated CABG (84.5%), valve surgeries (9.9%), and CABG + valve surgeries (5.6%) in addition to patients' cardiovascular risk factors from 2002 to 2021 at THC. The overall trend of all cardiovascular risk factors (i.e., diabetes, hypertension, hyperlipidemia, cigarette smoking, IHD family history, opium use, and BMI ≥ 30) was increasing in these years. Intriguingly, the prevalence of different risk factors among the three studied groups followed a similar pattern: the AS.AVR/r group fell in between the CABG and IVS groups for all risk factors. With an overall rate of 1.9% for in-hospital mortality, patients undergoing CABG + valve had the highest mortality rate (7.2%).

The number of surgical revascularization (CABG) is different among different regions of the world and at the country-to-country level [1, 11]. This may stem from percutaneous coronary intervention (PCI) rates, which can affect CABG rates [11]. For instance, Spain has the largest PCI-to-CABG ratio of 8.63:1 and 31% CABG procedures [12]. In comparison, the UK had a PCI-to-CABG ratio of 2.03:1 and 65% CABG percentage among the procedures [11]. In our study, 84.5% of operations were isolated CABG, which is higher in comparison with other European countries as well, such as 70% in Denmark and 12% in Norway [1]. Reports from the US STS registry also indicated that more than half of the procedures were isolated CABG [13]. Our CABG prevalence was congruent with previous THC databank reports in 2008 and 2012, reporting 87.2% and 78.8% isolated CABGs, respectively [5, 6].

An increase in AVR/r and MVR/r was observed based on our reports. Country-to-country differences also exist in the decision for valve operation, ranging from 57 to 73% within different European countries [14]. In Iran, transcatheter aortic valve replacement (TAVR) has not replaced surgical aortic valve replacement (SAVR), hence the substantial need for surgical valve replacements.

In our study, males were dominant in most of the procedures. This is in line with reports of the Global Burden of Disease Study 2019 on the CVDs [15]. There have been conflicting results in terms of sex differences in cardiac surgeries. It has been suggested that there is no male–female difference in the AV surgery [16–18]. However, a nationwide cohort study in the Netherlands reported a substantial difference in presentations and the predominance of males and females [19]. In terms of CABG, in a report by Flameng et al., the female sex in addition to older age was associated with early mortality in the concomitant valve and CABG surgery [20]. Regarding age, as another risk factor, CABG patients were the oldest group. This seems rational, as an increase in age is associated with an increase in other risk factors and comorbidities [21–23]. The fact that mean age had an increasing trend from 2002 to 2021 might be good news for Iran's healthcare system, as in recent years, people have stayed healthier for a longer period of time. Age was recognized as one of the top features associated with poor outcomes and mortality after CABG in another report from THC using machine learning methods [24]; hence, emphasizing the need for further focus. As expected, the prevalence of diabetes, hypertension, obesity, and smoking, as the classic ischemic heart disease risk factors, was highest in patients undergoing CABG in our study, all of which have been associated with poor prognosis in patients undergoing CABG [25–27].

We observed the prevalence of cardiovascular risk factors in the AS.AVR/r group lay between the CABG and IVS groups. A higher similarity between AS.AVR/r and CABG group than that between IVS and CABG group could be justified on the grounds of aging role in both coronary artery disease and severe AS. Severe AS is now perceived predominantly as a senile disease, and therefore it accompanies an increased risk of conventional cardiovascular risk factors, as does coronary artery diseases [28]. Furthermore, these two conditions share a similar inflammatory-atherosclerotic pathology; AS pathology involves the initiation of inflammation due to hemodynamic stress. This inflammation, in turn, facilitates the infiltration of lipids, ultimately leading to the establishment of calcification and valve leaflet immobility. Consequently, it has been shown that the primary AS plaque resembles that of coronary artery disease [28]. By the same token, medications that are prescribed routinely for coronary artery disease, particularly statins and angiotensin-converting enzyme inhibitors, have shown promising potential in slowing AS progression [29, 30]. However, their implications for AS disease should be systematically reviewed and confirmed in prospective clinical trials.

One of the most prominent findings of our study was the fact that most of the risk factors had increasing trends during this 20-year period in THC. In our center, a report from 2005–2015 on patients who underwent CABG showed that increasing trends were observed in BMI, hypertension prevalence, and diabetes [31]. Several studies have been conducted to assess temporal trends of cardiovascular risk factors in different countries. A serial cross-sectional study conducted in the US showed that among 50,571 individuals there were significant increases in BMI and hemoglobin A1c while serum total cholesterol and smoking were decreasing during a 20-year period [32]. In France, there was a significant decrease in hypertension and smoking rates from 2007 to 2012. Moreover, the 10-year Framingham risk score decreased from 13.3% to 11.7% in men and 8% to 5.9% in women [33]. In another study conducted on Swedish women during a 34-year period, an increase in BMI was observed while smoking diminished [34]. Differences in these trends mainly depend on racial and ethnic differences which have been well-studied [35, 36]. The difference might be attributed to changes in lifestyle and habitual patterns in Iran. Moreover, several changes in healthcare policies, demographic changes, and advancements in medical technologies. Also, it should be noted that unlike the studies mentioned, our population comprised of patients referred to THC for surgery which might be completely distinct from these normal populations. Among the limited number of studies conducted on patients undergoing CABG, a worsening trend in risk factors, such as hypertension and diabetes, was observed over time [37]. In a much larger cohort in the United States among patients undergoing CABG, increases in the rates of obesity, left main coronary artery disease, and advanced NYHA heart failure [38]. Strategies should target better lifestyle and also secondary prevention in patients with established cardiovascular disease. Some goals have also been determined by “Iran’s National Action Plan for Prevention and Control of Non-Communicable Diseases (NCDs)”. For instance, this plan indicated that salt intake should be reduced by 30% by 2025 [39]. Furthermore, in this national action plan, there are nine targets based on the global action plan for NCDs and four special targets regarding trans fatty acid, traffic injuries, drug abuse, and mental diseases [40]. Promisingly, the quality of diabetes care, as another main risk factor, has improved in Iran; however, there is still a long way to achieve the goals in this area [41].

Regarding in-hospital mortality, the highest rate was for CABG + valve procedures, the same as the data reported for the US and Europe [1, 13, 42]. Also, the STS 2021 report showed a declining trend for isolated AVR, MVR, and MV repair from 2010 to 2019, while our data showed increasing trends. Again, this might be attributed to a switch toward transcatheter procedures that are currently performed at a low rate in Iran. It should be noted that when comparing mortality rates between countries and regions, several factors, such as the mean age of patients in the database and rates of risk factors, should be considered. It seems that in recent years, the mortality rate for CABG has slightly increased, which was the main contributor to the overall increase in mortality in THC. The underlying reason might be the increase in overall age and risk factors mentioned earlier. Finally, the impact of the COVID-19 pandemic on the overall mortality of the procedures should be taken into consideration. In our data, the highest mortality rate was reported as 4.0% in 2020 which was the first year of the pandemic. This is in line with the findings of Wang et al. [43] that found a short-term increase in mortality from cardiac surgeries. This could be attributed to both pre-procedural and post-procedural COVID-19 infection [44].

Our findings have several clinical and healthcare policy implications. First, considering changes in risk factors during this period of time, clinicians can put a higher focus on newer risk factors and provide patients with better lifestyle modifications and pharmacological therapies aiming at reducing the risk of patients. Moreover, recognizing the patterns of surgeries in different regions could lead to better design of public health policies, tailored for a specific region and with localized risk factors. Plans such as Iran’s National Action Plan for Prevention and Control of Non-Communicable Diseases (NCDs) could be one of these plans. Similar strategies could be provided for patients undergoing cardiac surgeries, especially CABG.

The strengths of this study include but are not limited to large sample size, high surgery volume (3198 cases per year), and low missing values. However, there were some limitations. First, using data from a single center prevented us to generalize these findings to other centers and countries. Second, mortality rates between different cardiac surgeries may have been affected by baseline characteristics, type of surgery, disease severity, and other confounding variables. Third, there were several other related risk factors for which data was not available and could be considered another limitation. In the current study, we did not aim to assess the factors related to in-hospital mortality. Hence, further studies are warranted to investigate these associations in the THC database. Finally, due to the cross-sectional nature of this study, we were unable to provide causal relationships between trends of risk factors and the type of cardiac surgery.

## Conclusion

With the significant increase in cardiovascular diseases in recent years, the importance of determining appropriate policies to control this trend has increased. In the big picture, the THC cardiac surgery database is a good measure of trends in cardiac surgeries, especially in Iran. Monitoring the mortality trend, the number of surgeries, and the risk factors related to each surgery by this database and other similar databases in Iran and other countries can have an important contribution to policies to reduce the burden of cardiovascular diseases. Based on our findings, the rising trends of cardiovascular risk factors might have outpaced these preventive strategies.

### Supplementary Information

**Additional file 1:** **Supplementary Table 1.** Twenty-year trends of cardiac surgeries. **Supplementary Table 2. **Distribution of valve surgeries from 2002 to 2021. **Supplementary Table 3. **Distribution of aortic valve replacement and repair. **Supplementary Table 4. **Distribution of mitral valve replacement and repair. **Supplementary Table 5.** Twenty-year trends of cardiovascular risk factors according to cardiac surgery types. **Supplementary Table 6.** Twenty-year trends of in-hospital mortality among cardiac surgeries

## Data Availability

The data used in this study will be made available upon reasonable request from the corresponding author.
