# Supplementary material for: Heart surgery over two decades: what we have learned about results and changing risks
Source: BMC Cardiovasc Disord. 2024 Apr 5;24:195. doi: 10.1186/s12872-024-03860-9 (PMC10996112; doi:10.1186/s12872-024-03860-9)
Supplement: Supplementary file 1 — Additional file 1: Supplementary Table 1. Twenty-year trends of cardiac surgeries. Supplementary Table 2. Distribution of valve surgeries from 2002 to 2021. Supplementary Table 3. Distribution of aortic valve replacement and repair. Supplementary Table 4. Distribution of mitral valve replacement and repair. Supplementary Table 5. Twenty-year trends of cardiovascular risk factors according to cardiac surgery types. Supplementary Table 6. Twenty-year trends of in-hospital mortality among cardiac surgeries [file 12872_2024_3860_MOESM1_ESM.docx]

**Variable definitions**

**Hypertension** was defined as whether a) a history of treatment for hypertension; b) documented systolic blood pressure (SBP) and/or diastolic blood pressure (DBP) above the following certain levels on at least two occasions: b-1) SBP≥ 140 mmHg and/or DBP≥90 mmHg in the absence of diabetes or chronic kidney disease (CKD), b-2) SBP≥130 and/or DBP≥80 in the presence of diabetes or CKD; or c) current pharmacological treatment.

**Diabetes** was defined according to the American Diabetes Association criteria as whether a) hemoglobin A1c ≥6.5%; b) fasting plasma glucose ≥126 mg/dL ; c) 2-h plasma glucose ≥200 mg/dL (on glucose tolerance test); or d) a random plasma glucose ≥200 mg/dL in a patient with hyperglycemia symptoms.

**Hyperlipidemia** was defined as whether a) a history of or current diagnosis and/or treatment for hyperlipidemia or b) total cholesterol ≥200 mg/dL or low-density-lipoprotein cholesterol ≥130 mg/dL or triglyceride≥150 mg/dL.

**Current cigarette smoker** was defined as a person who have had smoked at least 100 cigarettes in total and has been smoking for at least one previous month.

**Opium ever use** was defined as current or past consumption of opium irrespective of the consumption method (smoking, ingestion, or intravenous use).

**Positive family history** **of ischemic heart disease** was defined as the occurrence of any of the followings among first-degree relatives (<65 year-old female or <55-year-old male relatives): a) sudden cardiac death; b) percutaneous coronary intervention; c) coronary artery bypass graft; or d) significant coronary stenosis

**Body mass index (BMI)** was calculated as “Weight (kg)/height^2^ (m^2^)” and patients were classified as normal weight/overweight (BMI<30) or obese (BMI≥30).

STROBE Statement—Checklist of items that should be included in reports of ***cross-sectional studies***

|  | Item No | Recommendation | Paragraph No |
| --- | --- | --- | --- |
| **Title and abstract** | 1 | (*a*) Indicate the study’s design with a commonly used term in the title or the abstract | Title |
|  |  | (*b*) Provide in the abstract an informative and balanced summary of what was done and what was found | Abstract |
| Introduction | | | |
| Background/rationale | 2 | Explain the scientific background and rationale for the investigation being reported | Introduction, P1-2 |
| Objectives | 3 | State specific objectives, including any prespecified hypotheses | Introduction, P2 |
| Methods | | | |
| Study design | 4 | Present key elements of study design early in the paper | Methods, Section 2.1 |
| Setting | 5 | Describe the setting, locations, and relevant dates, including periods of recruitment, exposure, follow-up, and data collection | Methods, Section 2.1 |
| Participants | 6 | (*a*) Give the eligibility criteria, and the sources and methods of selection of participants | Methods, Section 2.2 |
| Variables | 7 | Clearly define all outcomes, exposures, predictors, potential confounders, and effect modifiers. Give diagnostic criteria, if applicable | Methods, Section 2.3 |
| Data sources/ measurement | 8* | For each variable of interest, give sources of data and details of methods of assessment (measurement). Describe comparability of assessment methods if there is more than one group | Methods, Section 2.3 |
| Bias | 9 | Describe any efforts to address potential sources of bias |  |
| Study size | 10 | Explain how the study size was arrived at | NA |
| Quantitative variables | 11 | Explain how quantitative variables were handled in the analyses. If applicable, describe which groupings were chosen and why | Methods, Section 2.4 |
| Statistical methods | 12 | (*a*) Describe all statistical methods, including those used to control for confounding | Methods, Section 2.4 |
|  |  | (*b*) Describe any methods used to examine subgroups and interactions | Methods, Section 2.4 |
|  |  | (*c*) Explain how missing data were addressed | Methods, Section 2.4 |
|  |  | (*d*) If applicable, describe analytical methods taking account of sampling strategy | NA |
|  |  | (*e*) Describe any sensitivity analyses | NA |
| Results | | | |
| Participants | 13* | (a) Report numbers of individuals at each stage of study—eg numbers potentially eligible, examined for eligibility, confirmed eligible, included in the study, completing follow-up, and analysed | Results, Section 3.1, 3.2, 3.3, and 3.4 |
|  |  | (b) Give reasons for non-participation at each stage | NA |
|  |  | (c) Consider use of a flow diagram | Figure 1 |
| Descriptive data | 14* | (a) Give characteristics of study participants (eg demographic, clinical, social) and information on exposures and potential confounders | Results, Section 3.1 |
|  |  | (b) Indicate number of participants with missing data for each variable of interest | NA |
| Outcome data | 15* | Report numbers of outcome events or summary measures | Results, Section 3.2, 3.3, and 3.4 |
| Main results | 16 | (*a*) Give unadjusted estimates and, if applicable, confounder-adjusted estimates and their precision (eg, 95% confidence interval). Make clear which confounders were adjusted for and why they were included | Results, Section 3.2, 3.3, and 3.4 |
|  |  | (*b*) Report category boundaries when continuous variables were categorized | NA |
|  |  | (*c*) If relevant, consider translating estimates of relative risk into absolute risk for a meaningful time period | NA |
| Other analyses | 17 | Report other analyses done—eg analyses of subgroups and interactions, and sensitivity analyses | NA |
| Discussion | | | |
| Key results | 18 | Summarise key results with reference to study objectives | Discussion, P1 |
| Limitations | 19 | Discuss limitations of the study, taking into account sources of potential bias or imprecision. Discuss both direction and magnitude of any potential bias | Discussion, P7 |
| Interpretation | 20 | Give a cautious overall interpretation of results considering objectives, limitations, multiplicity of analyses, results from similar studies, and other relevant evidence | Conclusion |
| Generalisability | 21 | Discuss the generalisability (external validity) of the study results | Conclusion |
| Other information | | | |
| Funding | 22 | Give the source of funding and the role of the funders for the present study and, if applicable, for the original study on which the present article is based | Declarations section |

*Give information separately for exposed and unexposed groups.

**Note:** An Explanation and Elaboration article discusses each checklist item and gives methodological background and published examples of transparent reporting. The STROBE checklist is best used in conjunction with this article (freely available on the Web sites of PLoS Medicine at http://www.plosmedicine.org/, Annals of Internal Medicine at http://www.annals.org/, and Epidemiology at http://www.epidem.com/). Information on the STROBE Initiative is available at www.strobe-statement.org.

**Supplementary Table 1.** Twenty-year trends of cardiac surgeries

|  | Total | CABG | CABG+valve | Valve |
| --- | --- | --- | --- | --- |
| 2002 | 1322 | 1216(92.0%) | 22(1.7%) | 84(6.4%) |
| 2003 | 2549 | 2343(91.9%) | 61(2.4%) | 145(5.7%) |
| 2004 | 2811 | 2573(91.5%) | 76(2.7%) | 162(5.8%) |
| 2005 | 2963 | 2710(91.5%) | 88(3.0%) | 165(5.6%) |
| 2006 | 2921 | 2590(88.7%) | 128(4.4%) | 203(6.9%) |
| 2007 | 3107 | 2787(89.7%) | 115(3.7%) | 205(6.6%) |
| 2008 | 3379 | 2960(87.6%) | 172(5.1%) | 247(7.3%) |
| 2009 | 3109 | 2670(85.9%) | 189(6.1%) | 250(8.0%) |
| 2010 | 2826 | 2382(84.3%) | 198(7.0%) | 246(8.7%) |
| 2011 | 2559 | 2140(83.6%) | 194(7.6%) | 225(8.8%) |
| 2012 | 2436 | 2013(82.6%) | 173(7.1%) | 250(10.3%) |
| 2013 | 2341 | 1947(83.2%) | 161(6.9%) | 233(10%) |
| 2014 | 3278 | 2716(82.9%) | 187(5.7%) | 375(11.4%) |
| 2015 | 3332 | 2682(80.5%) | 220(6.6%) | 430(12.9%) |
| 2016 | 3267 | 2591(79.3%) | 223(6.8%) | 453(13.9%) |
| 2017 | 2925 | 2265(77.4%) | 215(7.4%) | 445(15.2%) |
| 2018 | 2837 | 2257(79.6%) | 171(6.0%) | 409(14.4%) |
| 2019 | 2598 | 2089(80.4%) | 174(6.7%) | 335(12.9%) |
| 2020 | 1832 | 1418(77.4%) | 152(8.3%) | 262(14.3%) |
| 2021 | 2026 | 1620(80%) | 148(7.3%) | 258(12.7 %) |
| Data are presented as frequency (percentages).  CABG: coronary artery bypass graft | | | | |

**Supplementary Table 2.** Distribution of valve surgeries from 2002 to 2021

|  | Total | AVR/r | MVR/r | Others |
| --- | --- | --- | --- | --- |
| 2002 | 84 | 20 (23.81%) | 24 (28.57%) | 40 (47.62%) |
| 2003 | 145 | 41 (28.28%) | 38 (26.21%) | 66 (45.52%) |
| 2004 | 162 | 38 (23.46%) | 38 (23.46%) | 86 (53.09%) |
| 2005 | 165 | 37 (22.42%) | 50 (30.3%) | 78 (47.27%) |
| 2006 | 203 | 41 (20.2%) | 62 (30.54%) | 100 (49.26%) |
| 2007 | 205 | 46 (22.44%) | 71 (34.63%) | 88 (42.93%) |
| 2008 | 247 | 58 (23.48%) | 76 (30.77%) | 113 (45.75%) |
| 2009 | 250 | 66 (26.4%) | 74 (29.6%) | 110 (44%) |
| 2010 | 246 | 63 (25.61%) | 75 (30.49%) | 108 (43.9%) |
| 2011 | 225 | 55 (24.44%) | 48 (21.33%) | 122 (54.22%) |
| 2012 | 250 | 70 (28%) | 70 (28%) | 110 (44%) |
| 2013 | 233 | 57 (24.46%) | 71 (30.47%) | 105 (45.06%) |
| 2014 | 375 | 86 (22.93%) | 70 (18.67%) | 219 (58.4%) |
| 2015 | 430 | 107 (24.88%) | 141 (32.79%) | 182 (42.33%) |
| 2016 | 453 | 154 (34%) | 141 (31.13%) | 158 (34.88%) |
| 2017 | 445 | 147 (33.03%) | 142 (31.91%) | 156 (35.06%) |
| 2018 | 409 | 117 (28.61%) | 142 (34.72%) | 150 (36.67%) |
| 2019 | 335 | 118 (35.22%) | 104 (31.04%) | 113 (33.73%) |
| 2020 | 262 | 97 (37.02%) | 92 (35.11%) | 73 (27.86%) |
| 2021 | 258 | 75 (29.07%) | 129 (50%) | 54 (20.93%) |
| Data are presented as frequency (percentages).  AVR/r: aortic valve replacement/repair  MVR/r: mitral valve replacement/repair | | | | |

**Supplementary Table 3.** Distribution of aortic valve replacement and repair

|  | Total | AVR | AVr |
| --- | --- | --- | --- |
| 2002 | 20 | 20 (100%) | 0 (0%) |
| 2003 | 41 | 41 (100%) | 0 (0%) |
| 2004 | 38 | 36 (95%) | 2 (5%) |
| 2005 | 37 | 37 (100%) | 0 (0%) |
| 2006 | 41 | 41 (100%) | 0 (0%) |
| 2007 | 46 | 46 (100%) | 0 (0%) |
| 2008 | 58 | 57 (97.9%) | 1 (2.1%) |
| 2009 | 66 | 66 (100%) | 0 (0%) |
| 2010 | 63 | 62 (98.5%) | 1 (1.5%) |
| 2011 | 55 | 54 (98.4%) | 1 (1.6%) |
| 2012 | 70 | 70 (100%) | 0 (0%) |
| 2013 | 57 | 54 (94.5%) | 3 (5.5%) |
| 2014 | 86 | 83 (96.6%) | 3 (3.4%) |
| 2015 | 107 | 100 (93.5%) | 7 (6.5%) |
| 2016 | 154 | 142 (92%) | 12 (8%) |
| 2017 | 147 | 145 (98.6%) | 2 (1.4%) |
| 2018 | 117 | 115 (98.3%) | 2 (1.7%) |
| 2019 | 118 | 114 (96.7%) | 4 (3.3%) |
| 2020 | 97 | 95 (97.4%) | 2 (2.6%) |
| 2021 | 75 | 72 (96.6%) | 3 (3.4%) |

Data are presented as frequency (percentages).

AVR: aortic valve replacement

AVr: aortic valve repair

**Supplementary Table 4.** Distribution of mitral valve replacement and repair

|  | Total | MVR | MVr |
| --- | --- | --- | --- |
| 2002 | 24 | 22 (91.7%) | 2 (8.3%) |
| 2003 | 38 | 35 (92.1%) | 3 (7.9%) |
| 2004 | 38 | 37 (97.4%) | 1 (2.6%) |
| 2005 | 50 | 50 (100%) | 0 (0%) |
| 2006 | 62 | 57 (91.9%) | 5 (8.1%) |
| 2007 | 71 | 67 (94.4%) | 4 (5.6%) |
| 2008 | 76 | 71 (93.4%) | 5 (6.6%) |
| 2009 | 74 | 64 (86.7%) | 10 (13.3%) |
| 2010 | 75 | 60 (79.7%) | 15 (20.3%) |
| 2011 | 48 | 40 (83.3%) | 8 (16.7%) |
| 2012 | 70 | 62 (88.4%) | 8 (11.6%) |
| 2013 | 71 | 55 (77.5%) | 16 (22.5%) |
| 2014 | 70 | 59 (84.1%) | 11 (15.9%) |
| 2015 | 141 | 114 (80.6%) | 27 (19.4%) |
| 2016 | 141 | 115 (81.3%) | 26 (18.7%) |
| 2017 | 142 | 133 (93.6%) | 9 (6.4%) |
| 2018 | 142 | 130 (91.4%) | 12 (8.6%) |
| 2019 | 104 | 95 (91.3%) | 9 (8.7%) |
| 2020 | 92 | 89 (96.7%) | 3 (3.3%) |
| 2021 | 129 | 128 (99.2%) | 1 (0.8%) |

Data are presented as frequency (percentages).

MVR: mitral valve replacement

MVr: mitral valve repair

**Supplementary Table 5.** Twenty-year trends of cardiovascular risk factors according to cardiac surgery types

|  |  | Age | Diabetes | Hypertension | Current cigarette Smoking | Opium ever use | LVEF<40 | BMI≥30 |
| --- | --- | --- | --- | --- | --- | --- | --- | --- |
| CABG (N=45969) | 2002-2021 | 63.37±10.33 | 17386(37.9%) | 24839(54.1%) | 8594(18.7%) | 5853(14.9%) | 13485(29.6%) | 10138(21.6%) |
|  | 2002 | 57.72±9.54 | 338(27.8%) | 664(54.6%) | 387(31.9%) | 27(10.8%) | 276(23.5%) | 209(17.2%) |
|  | 2003 | 58.34±9.88 | 678(28.9%) | 1021(43.6%) | 481(20.6%) | 48(9.1%) | 638(27.7%) | 504(21.5%) |
|  | 2004 | 58.35±9.58 | 735(28.6%) | 1124(43.7%) | 352(13.7%) | 95(9.7%) | 673(26.6%) | 566(22.0%) |
|  | 2005 | 58.93±9.43 | 865(31.9%) | 1703(62.8%) | 368(13.6%) | 201(11.6%) | 666(24.8%) | 563(20.8%) |
|  | 2006 | 58.86±9.25 | 917(35.4%) | 1514(58.5%) | 384(14.8%) | 303(13.2%) | 611(23.8%) | 596(23.1%) |
|  | 2007 | 59.29±9.57 | 935(33.5%) | 1476(53%) | 327(11.7%) | 417(15.0%) | 598(21.6%) | 691(24.8%) |
|  | 2008 | 59.84±9.64 | 955(32.3%) | 1423(48.1%) | 587(19.8%) | 326(11.0%) | 723(24.6%) | 711(24.1%) |
|  | 2009 | 66.38±10.80 | 979(36.7%) | 1480(55.4%) | 454(17%) | 305(11.4%) | 678(25.5%) | 585(22.1%) |
|  | 2010 | 70.12±9.32 | 883(37.1%) | 1226(51.5%) | 371(15.6%) | 339(14.2%) | 650(27.4%) | 521(22.0%) |
|  | 2011 | 69.27±9.43 | 884(41.3%) | 1005(47%) | 323(15.1%) | 340(15.9%) | 717(33.5%) | 435(20.4%) |
|  | 2012 | 68.51±9.71 | 803(39.9%) | 872(43.3%) | 255(12.7%) | 252(12.5%) | 576(28.6%) | 430(21.4%) |
|  | 2013 | 67.16±9.41 | 795(40.8%) | 1067(54.8%) | 382(19.6%) | 323(16.6%) | 570(29.3%) | 474(24.4%) |
|  | 2014 | 66.26±9.59 | 1139(42.0%) | 1642(60.5%) | 508(18.8%) | 510(18.8%) | 877(32.4%) | 690(25.6%) |
|  | 2015 | 65.72±9.42 | 1121(41.8%) | 1595(59.5%) | 523(19.6%) | 492(18.3%) | 892(33.3%) | 658(24.66%) |
|  | 2016 | 65.18±9.63 | 1052(40.6%) | 1443(55.7%) | 583(22.6%) | 477(18.4%) | 860(33.3%) | 620(24.3%) |
|  | 2017 | 65.39±9.82 | 979(43.7%) | 1228(54.8%) | 515(23.0%) | 293(16.3%) | 744(33.9%) | 317(23.1%) |
|  | 2018 | 64.42±9.34 | 1002(44.8%) | 1275(57%) | 514(23.0%) | 302(15.3%) | 822(37.5%) | 462(26.9%) |
|  | 2019 | 63.31±9.31 | 961(46.1%) | 1212(58.2%) | 489(23.5%) | 281(14.4%) | 834(40.6%) | 452(25.7%) |
|  | 2020 | 62.13±8.79 | 649(46%) | 836(59.3%) | 324(23.0%) | 232(16.9%) | 548(39.2%) | 333(25.6%) |
|  | 2021 | 62.46±8.94 | 716(44.4%) | 1033(64.1%) | 467(29.0%) | 290(20.6%) | 532(33.5%) | 321(28.5%) |
| AS.AVR/r (N=909) | 2002-2021 | 59.70±15.55 | 139(16.1%) | 311(36.0%) | 115(13.3%) | 113(14.6%) | 156(18%) | 159(23.1%) |
|  | 2002 | 51.40±14.21 | 0(0.0%) | 3(30.00%) | 1(10%) | 0(0%) | 1(10.0%) | 1(10.0%) |
|  | 2003 | 58.17±17.58 | 1(5.9%) | 7(41.2%) | 4(23.5%) | 0(0%) | 3(16.0%) | 1(5.9%) |
|  | 2004 | 51.24±15.49 | 2(9.5%) | 3(14.3%) | 4(19%) | 0(0%) | 4(19.1%) | 5(23.8%) |
|  | 2005 | 56.67±18.52 | 4(12.9%) | 11(35.5%) | 5(16.1%) | 0(0%) | 5(16.12%) | 4(12.9%) |
|  | 2006 | 50.46±15.03 | 2(7.4%) | 12(44.4%) | 5(18.5%) | 2(8.0%) | 2(7.421%) | 3(11.5%) |
|  | 2007 | 53.31±15.94 | 2(8.0%) | 8(32%) | 3(12%) | 3(12.0%) | 7(28.0%) | 5(20.8%) |
|  | 2008 | 51.88±17.30 | 2(6.7%) | 11(36.7%) | 3(10%) | 1(3.3%) | 4(13.8%) | 7(24.1%) |
|  | 2009 | 61.95±15.76 | 1(4.5%) | 9(40.9%) | 4(18.2%) | 5(22.7%) | 4(18.9%) | 4(19.1%) |
|  | 2010 | 65.97±16.72 | 7(23.3%) | 5(16.7%) | 2(6.7%) | 4(13.3%) | 6(20.0%) | 2(6.9%) |
|  | 2011 | 65.33±16.13 | 6(23.1%) | 8(30.8%) | 3(11.5%) | 7(26.9%) | 3(11.5%) | 3(12.5%) |
|  | 2012 | 65.79±14.09 | 7(18.4%) | 8(21.1%) | 1(2.6%) | 5(13.2%) | 7(18.4%) | 9(25.0%) |
|  | 2013 | 50.18±17.44 | 3(10.7%) | 5(17.9%) | 1(3.6%) | 1(3.6%) | 3(11.1%) | 5(19.2%) |
|  | 2014 | 60.93±14.13 | 4(7.4%) | 20(37%) | 7(13%) | 12(22.2%) | 9(16.7%) | 20(37.0%) |
|  | 2015 | 58.04±16.26 | 15(21.4%) | 21(30%) | 11(15.7%) | 10(14.3%) | 18(25.7%) | 22(32.4%) |
|  | 2016 | 62.79±16.66 | 21(25.6%) | 29(35.4%) | 9(11%) | 11(13.1%) | 11(13.8%) | 14(25.94%) |
|  | 2017 | 60.84±14.17 | 13(13.3%) | 35(35.7%) | 18(18.4%) | 17(22.1%) | 17(17.4%) | 9(18.4%) |
|  | 2018 | 62.6±13.86 | 15(20.5%) | 32(43.8%) | 8(11%) | 9(14.5%) | 11(15.9%) | 9(24.34%) |
|  | 2019 | 60.32±12.39 | 14(18.4%) | 37(48.7%) | 18(23.7%) | 9(13.4%) | 15(20.6%) | 16(32.7%) |
|  | 2020 | 61.3±13.98 | 11(18.3%) | 29(48.3%) | 2(3.3%) | 11(18.6%) | 14(23.7%) | 12(21.1%) |
|  | 2021 | 58.69±11.22 | 9(20.0%) | 18(40%) | 6(13.3%) | 6(14.6%) | 12(26.7%) | 8(25.8%) |
| IVS (N=4473) | 2002-2021 | 54.55±13.88 | 522(11.8%) | 1276(28.9%) | 309(7. 0%) | 239(0.6%) | 723(16.6%) | 809(19.8%) |
|  | 2002 | 48.23±13.86 | 9(12.2%) | 29(39.2%) | 7(9.5%) | 0(0%) | 10(13.7%) | 11(14.9%) |
|  | 2003 | 47.57±13.36 | 11(8.7%) | 22(17.3%) | 9(7.1%) | 1(1.8%) | 19(15.7%) | 14(11.1%) |
|  | 2004 | 46.82±14.03 | 9(6.3%) | 18(12.7%) | 8(5.6%) | 0(0%) | 18(13.3%) | 20(14.1%) |
|  | 2005 | 49.82±12.07 | 15(11.2%) | 33(24.6%) | 13(9.7%) | 2(2.8%) | 16(12.1%) | 12(9.0%) |
|  | 2006 | 49.01±12.52 | 14(8.0%) | 47(26.7%) | 9(5.1%) | 11(6.7%) | 23(13.4%) | 29(16.5%) |
|  | 2007 | 48.20±13.57 | 16(8.9%) | 41(22.8%) | 8(4.4%) | 11(6.1%) | 25(13.9%) | 27(15.1%) |
|  | 2008 | 49.33±13.20 | 28(12.8%) | 60(27.5%) | 21(9.6%) | 12(5.5%) | 36(16.8%) | 33(15.1%) |
|  | 2009 | 55.46±13.48 | 26(11.8%) | 53(24.0%) | 14(6.3%) | 8(3.6%) | 33(14.9%) | 37(16.8%) |
|  | 2010 | 57.79±14.68 | 30(14.2%) | 54(25.5%) | 15(7.1%) | 12(5.7%) | 41(19.4%) | 34(16.1%) |
|  | 2011 | 58.58±13.77 | 22(11.4%) | 50(25.9%) | 14(7.3%) | 9(4.7%) | 33(17.1%) | 36(18.7%) |
|  | 2012 | 58.06±12.97 | 18(8.7%) | 56(27.2%) | 9(4.4%) | 7(3.4%) | 22(10.7%) | 38(18.5%) |
|  | 2013 | 56.06±13.56 | 15(7.5%) | 60(29.9%) | 15(7.5%) | 17(8.5%) | 31(15.4%) | 37(18.4%) |
|  | 2014 | 56.93±13.56 | 36(11.3%) | 98(30.7%) | 13(4.1%) | 20(6.3%) | 56(17.6%) | 81(25.5%) |
|  | 2015 | 56.37±13.18 | 43(12.4%) | 123(35.4%) | 23(6.6%) | 28(8.1%) | 75(21.6%) | 82(23.8%) |
|  | 2016 | 55.82±13.86 | 40(11.7%) | 98(28.7%) | 26(7.6%) | 26(7.6%) | 58(16.9%) | 66(19.9%) |
|  | 2017 | 57.01±13.43 | 44(13.1%) | 101(30.1%) | 30(8.9%) | 14(5%) | 49(14.8%) | 56(25.0%) |
|  | 2018 | 55.66±13.77 | 45(13.6%) | 102(30.8%) | 21(6.3%) | 9(3.1%) | 48(14.9%) | 60(24.6%) |
|  | 2019 | 56.31±12.98 | 36(14.5%) | 95(38.3%) | 12(4.8%) | 16(7.2%) | 48(19.7%) | 47(24.1%) |
|  | 2020 | 54.45±13.75 | 35(17.8%) | 65(33.0%) | 18(9.1%) | 17(8.9%) | 42(21.3%) | 44(23.5%) |
|  | 2021 | 55.42±12.82 | 30(14.7%) | 71(34.8%) | 24(11.8%) | 19(10.1%) | 40(19.7%) | 45(26.5%) |
| Data are presented as frequency (percentages) or mean±SD.  CABG: coronary artery graft, LVEF: left-ventricular ejection fraction, BMI: body mass index , AS: Aortic stenosis, AI: aortic insufficiency, AVR/r: Aortic valve replacement/repair | | | | | | | | |

**Supplementary Table 6.** Twenty-year trends of in-hospital morality among cardiac surgeries

| Year | Total | CABG | CABG+valve | Valve |
| --- | --- | --- | --- | --- |
| 2002-2021 | 1046(1.9%) | 634 (1.4%) | 222 (7.2%) | 190 (3.5%) |
| 2002 | 26(2.0%) | 16 (1.3%) | 2 (9.1%) | 8 (9.5%) |
| 2003 | 31(1.2~~)~~ | 16 (0.7%) | 7 (11.5%) | 8 (5.5%) |
| 2004 | 26(0.9%) | 14 (0.5%) | 7 (9.2%) | 5 (3.1%) |
| 2005 | 27(0.9%) | 20 (0.7%) | 2 (2.3%) | 5 (3.0%) |
| 2006 | 45(0.5%) | 23 (0.9%) | 8 (6.3%) | 14 (6.9%) |
| 2007 | 51(1.6%) | 36 (1.3%) | 10 (8.7%) | 5 (2.4%) |
| 2008 | 54(1.6%) | 29 (1.0%) | 16 (9.3%) | 9 (3.6%) |
| 2009 | 43(1.4%) | 20 (0.8%) | 13 (6.9%) | 10 (4.0%) |
| 2010 | 67(2.4%) | 40 (1.7%) | 21 (10.6%) | 6 (2.4%) |
| 2011 | 50(2.0%) | 32 (1.5%) | 10 (5.2%) | 8 (3.6%) |
| 2012 | 37(1.5%) | 24 (1.2%) | 6 (3.5%) | 7 (2.8%) |
| 2013 | 38(1.6%) | 23 (1.2%) | 11 (6.8%) | 4 (1.7%) |
| 2014 | 64(2.0%) | 44 (1.6%) | 12 (6.4%) | 8 (2.1%) |
| 2015 | 83(2.5%) | 43 (1.6%) | 18 (8.2%) | 22 (5.1%) |
| 2016 | 55(1.7%) | 25 (1.0%) | 11 (4.9%) | 19 (4.2%) |
| 2017 | 61(2.1%) | 38 (1.7%) | 11 (5.1%) | 12 (2.7%) |
| 2018 | 69(2.4%) | 43 (1.9%) | 14 (8.2%) | 12 (2.9%) |
| 2019 | 75(2.9%) | 55 (2.6%) | 9 (5.2%) | 11 (3.3%) |
| 2020 | 73(4.0%) | 48 (3.4%) | 14 (9.2%) | 11 (4.2%) |
| 2021 | 71(3.5%) | 45 (2.8%) | 20 (13.5%) | 6 (2.3%) |
| Data are presented as frequency (percentages.  CABG: Coronary artery bypass graft | | | | |
